# In this Issue: Volume 113, Issue 1, January 2022

**DOI:** 10.1111/cas.14944

**Published:** 2022-01-10

## SNHG17, as an EMT‐related lncRNA, promotes the expression of c‐Myc by binding to c‐Jun in esophageal squamous cell carcinoma

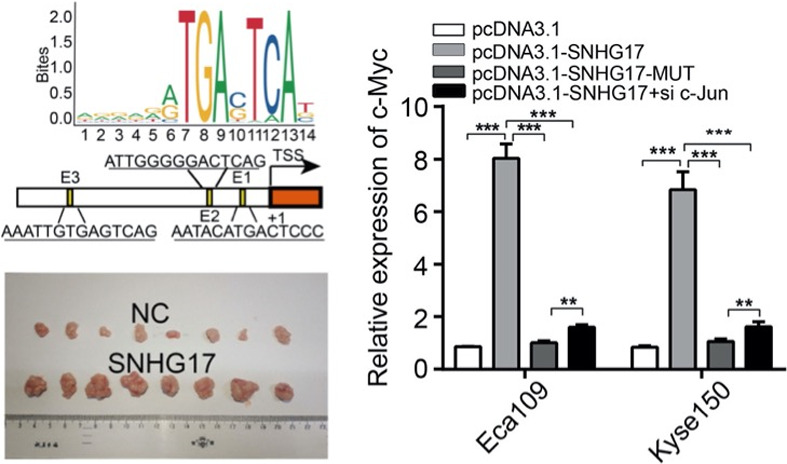

Long noncoding RNAs (lncRNAs) have been shown to regulate many cellular processes including cell proliferation, angiogenesis, and epithelial‐mesenchymal transition (EMT). SNHG17 is a lncRNA that has been found to be elevated in many cancers like non‐small cell lung adenocarcinoma and esophageal squamous cell carcinoma. In this study, Shen et al investigated the role of SNHG17 in inducing EMT in ESCC. They found that SNHG17 was elevated when an esophageal squamous cell carcinoma cell line was treated with TGFβ1, suggesting that SNHG17 contributes to TGFβ1‐induced EMT. Further experiments showed that SNHG17 recruited c‐Jun and this led to induction of c‐Myc, which is well known to participate in tumorigenesis. This SNHG17/c‐Jun/c‐Myc axis may be a potential therapeutic target.

https://onlinelibrary.wiley.com/doi/full/10.1111/cas.15184

## Hsa_circ_0005576 promotes osimertinib resistance through the miR‐512‐5p/IGF1R axis in lung adenocarcinoma cells

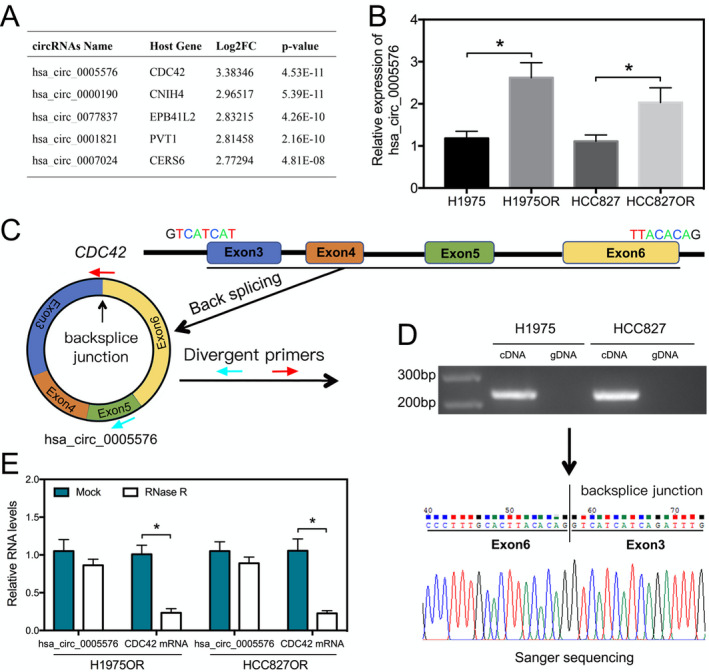

Epidermal growth factor receptor tyrosine kinase inhibitors (EGFR‐TKIs) have provided significant benefits over traditional chemotherapies. However, they are not a solution for lung cancer patients as the drug resistance invariably develops. The mechanisms for the drug resistance have been well studied, but 50% of the resistance develops via an unknown mechanism. In this study, Liu et al examined the role that hsa_circ_0005576 plays in generating resistance to osimertinib, a third generation EGFR‐TKI. The group showed that knockdown of hsa_circ_0005576 reversed the resistance of lung cancer cells to osimertinib. Pull down assays from the resistant cells showed miR‐512‐5p enrichment along with the hsa_circ_0005576. By sequestering miR‐512‐5p, hsa_circ_0005576 upregulated IGF1R signaling, which increases cell proliferation and prevents apoptosis. hsa_circ_0005576 may be a fruitful target to overcome osimertinib resistance in some lung adenocarcinoma patients.

https://onlinelibrary.wiley.com/doi/full/10.1111/cas.15177

## A novel function of HRP‐3 in regulating cell cycle progression via the HDAC‐E2F1‐Cyclin E pathway in lung cancer

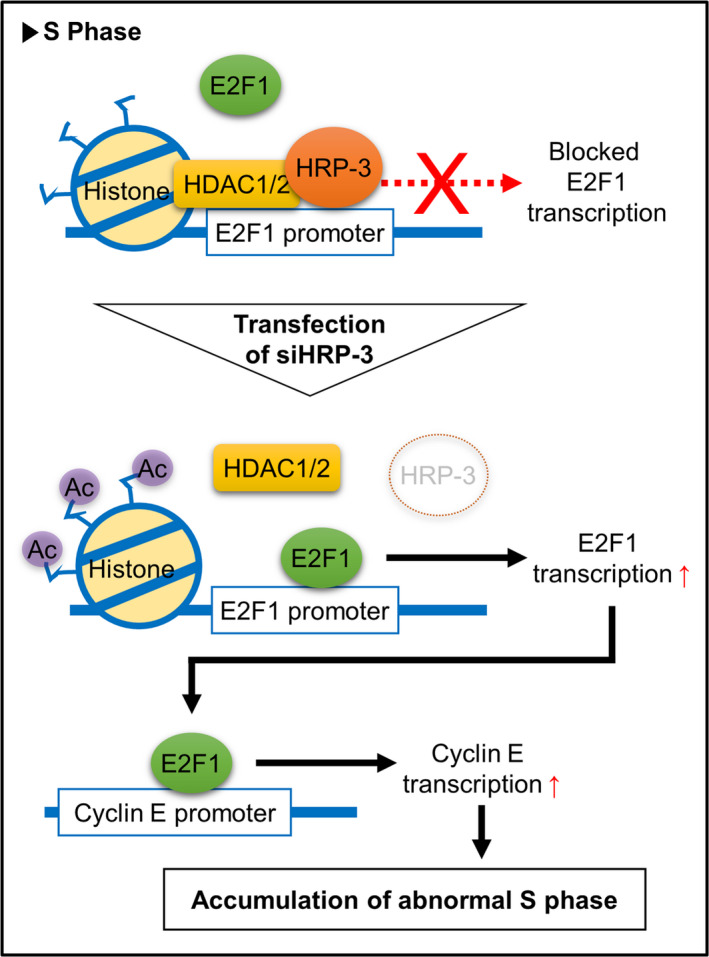

Lung cancer remains the leading cause of cancer death in both men and women. New biomarkers and therapeutics are needed to improve the clinical outcomes of these patients. In this study, Yun et al examined hepatoma‐derived growth factor (HDFG)‐related protein‐3 (HRP‐3), a protein that induces cell death in lung cancer cells. They found that patients with elevated levels of HRP‐3 had poor response to radiation and chemotherapy and had a worse prognosis. Further investigation showed that HRP‐3 recruited HDAC1/2 to increase acetylation of the E2F1 promotor. This epigenetic modification led to increased E2F1 and cyclin E with subsequent accumulation of cells in the S phase. Further study of this pathway may lead to improved care for cancer patients.

https://onlinelibrary.wiley.com/doi/full/10.1111/cas.15183

